# Health inequalities and development plans in Iran; an analysis of the past three decades (1984–2010)

**DOI:** 10.1186/1475-9276-13-42

**Published:** 2014-05-27

**Authors:** Hossein Zare, Antonio J Trujillo, Julia Driessen, Mojtaba Ghasemi, Gisselle Gallego

**Affiliations:** 1 Health Policy and Management Department, Johns Hopkins Bloomberg School of Public Health, 624 North Broadway, Baltimore, Maryland 21205, USA; 2 Department of International Health, Johns Hopkins Bloomberg School of Public Health, 615 North Wolfe Street, Baltimore, Maryland 21205, USA; 3 Hospital Management Research Center, Iran University of Medical Sciences, Tehran, Iran; 4 Graduate School of Public Health, University of Pittsburgh, 130 DeSoto Street A614 Crabtree, Pittsburgh, PA 15261, USA; 5 Department of Economics, University of Siena, Piazza San Francesco, 7, Siena, Italy; 6 Centre for Health Research, School of Medicine, University of Western Sydney, Sydney, New South Wales, Australia; 7 Faculty of Health Sciences, University of Sydney, Sydney, New South Wales, Australia

**Keywords:** Inequality, Health services, Health inequalities, Gini, Development plans, Kakwani, Gender inequalities, Out-of-pocket

## Abstract

**Introduction:**

Reducing inequalities in health care is one of the main challenges in all countries. In Iran as in other oil-exporting upper middle income countries, we expected to witness fewer inequalities especially in the health sector with the increase in governmental revenues.

**Methods:**

This study presents an inequalities assessment of health care expenditures in Iran. We used data from the Household Income and Expenditure Survey (HIES) in Iran from 1984–2010. The analysis included 308,735 urban and 342,532 rural households.

**Results:**

The results suggest heightened inequality in health care expenditures in Iran over the past three decades, including an increase in the gap between urban and rural areas. Furthermore, inflation has affected the poor more than the rich. The Kakwani progressivity index in all years is positive, averaging 0.436 in rural and 0.470 in urban areas during the time period of analysis. Compared to inequality in income distribution over the last 30 years, health expenditures continuously show more inequality and progressivity over the same period of time.

**Conclusions:**

According to the result of our study, during this period Iran introduced four National Development Plans (NDPs); however, the NDPs failed to provide sustainable strategies for reducing inequalities in health care expenditures. Policies that protect vulnerable groups should be prioritized.

## Introduction

Health care is a fundamental human right and one of the first responsibilities of a government is to establish effective and sustainable interventions to address inequality in health care [1] and protect low-income, vulnerable groups such as women and children, the elderly and the chronically ill [2]. In this regard, health care policymakers have long been concerned with protecting people from ill health that could lead to catastrophic household payments [3], which often push households into poverty [4].

Studies show that lower-income households have higher rates of catastrophic expenditures than higher-income groups [5,6]. These expenditures are shown to be one of the major factors compromising equitable health care systems in low-income countries [3,7]. The increase in health care expenditures coupled with high inflation rates—especially in developing countries—has increased out-of-pocket spending for health care, forced people to reject or quit treatment, and impacted household living standards [7]. The most devastating consequences of catastrophic payments are realized by those who are already poor, and who have to limit their expenditures to basic necessities such as food, and housing in order to afford health care [8]. Although there is no universal strategy for reducing catastrophic payments, universal health insurance [9], social protection systems [1], and exclusion of vulnerable groups from high cost-sharing payments are common strategies for protecting vulnerable populations [2] from the consequences of catastrophic payments [10].

Iran is a resource-rich country whose economy is highly dependent on crude oil and, like other oil-exporting countries, relies on these export revenues for economic growth [11]; more than 70% of annual state finances and more than 80% of annual foreign-exchange earnings are from the export of crude oil [12]. Countries like Iran are said to be “cursed” by natural resources because the resource bonanza does not translate into economic growth or development of wealth due to the country’s inability to successfully convert its depleting exhaustible resources into productive capital such as roads, buildings or health care systems [13]. The challenge for Iran and similar oil-producing countries is how to promote economic growth and develop wealth while reducing inequality among its citizens. As a country grows wealthier, it has more resources to spend on health care; however, studies and national health accounts have shown that resources allocated to the health care sector in Iran have been inadequate [14-17]. In recent years, Iran was expected to improve financial fairness and reduce economic barriers to accessing timely care for households facing catastrophic health events [16]. To this end, Iran rolled out its first five year economic, social, and cultural development plan (NDP) in 1989 [18]. Subsequent plans outlined the government’s intention to further invest in infrastructure and manage the macro-economy [19].

The first NDP (1990–1994) focused on development of primary health care (PHC) networks and medical facilities, especially in rural areas, and improving policies around human resources, family planning, and population control [18]. Developing a universal health insurance scheme was the main objective of the second NDP (1994–1999). The government established the Medical Services Insurance Organization (MSIO) in 1995 under the provisions of the Universal Health Insurance Act to provide medical insurance for civil servants, the needy, villagers and other social strata [20].

The third NDP (2000–2004) focused more on developing health in Iran under a “welfare state” approach [21], including improving both quality and quantity of coverage in rural areas, developing inpatient coverage for urban low-income uninsured people and reducing the cost of drugs for those with chronic renal failure, hemophila and thalassemia [22].

The Ministry of Welfare and Social Security (MWSS) was established in the last year of the third NDP. The stated primary objective of the fourth NDP (2005–2009) was decreasing inequalities in health expenditures.

All of Iran’s NDPs targeted economic inequality by prioritizing rural and lower-income groups, [18,21,23,24] and the then-leader (Ayatollah Khomeini) promised large-scale redistributions of wealth and income [19]. A 2009 comprehensive picture of poverty and income inequality in Iran provided by Salehi-Isfahani concluded that, in spite of declining poverty and inequality immediately following the revolution in Iran, income inequalities had increased in recent years [19]. Despite the NDP’s focus on improving health care access and equality, to date there are no studies evaluating the trend in health care expenditure inequality during the period of Iran’s four NDPs. The main contribution of this paper is to describe the trends in health care expenditure inequality in Iran during the last three decades (1984–2010).

### Iran’s health system

The total population of Iran was approximately 50 million in 1986, and increased to 75 million by 2011 [25]. During this time, the percentage of the population residing in rural areas fell from 45.7 percent to 28.5 percent [25]. In 2008 total expenditures on health care comprised 7.8% of Gross Domestic Product (GDP) [17].

The Ministry of Health and Medical Education (MOHME) acts as the main steward of the health care system. Nearly all of the primary and more than 71% of secondary and tertiary hospitals and rehabilitation facilities are public; the remaining 29% are private or nongovernmental organizations (NGOs) [26]. The proportion of NGO facilities is less than two percent [26]. Financing of health care in Iran is a combination of public funds (governmental budget), social health insurance, private insurance premiums and out-of-pocket payments.

**Figure 1:**
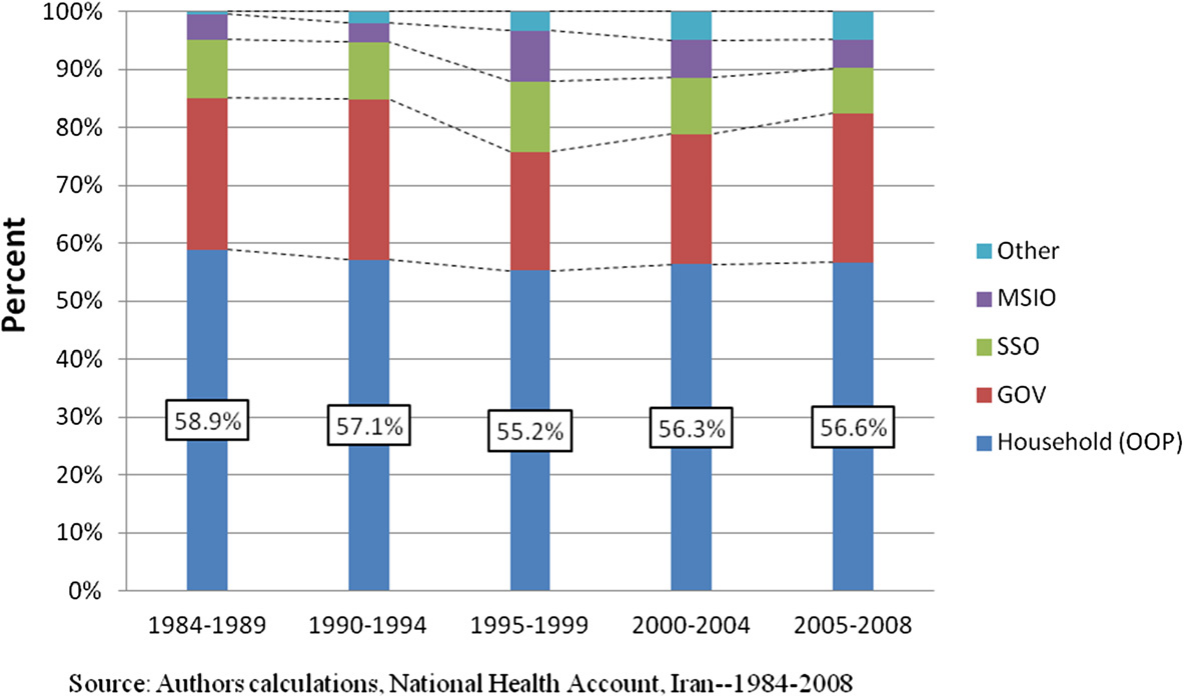
Comparing health care financing in four NDPs Iran.

Iranian insurers receive insurance premium revenue from their members as well as government support from general tax revenue and the sale of natural resources (mainly oil) [27]. Although more than 90% of people are covered by some type of public insurance, social health insurance or private insurance, the National Health Account showed that around 56.8% of total health care expenditures are financed out-of-pocket [14,28]. Figure 1 shows the breakdown in health care expenditures by payer from 1984–2008. During this time the proportion of expenditures that were out-of-pocket ranged from 55.2% to 58.9%. Household expenditure (Out-of-pocket payments) include household premium payments as well as direct payments for services. The lowest proportion of out-of-pocket spending was observed during the period of the second NDP. On average the government paid less than 25% (24.6%) from 1984–2008, with their largest contribution occurring during the second NDP. The Social Security Organization (SSO) was responsible for as much as 10% of health expenditures during this time. The MSIO as the main government health insurance organization represented on average 5.6% of health expenditures, peaking during the second NDP at 8.8%. The “Other” group in Figure 1 includes private health insurance companies introduced as part of the health insurance market, which has more recently increased its payer presence in the health care system.

## Methods

### Data sources

Data used for this study came from Iran’s Households Income and Expenditure Survey (HIES) from 1984 to 2010. The HIES is a nationally and regionally representative household survey conducted annually by the Statistical Center of Iran (SCI). It collects basic demographic and economic characteristics of households. Data on expenditures, wages, and income are self-reported. We drew on micro-data sets of HIES for the years 1984 to 2010 [17]. Households are chosen for inclusion in the survey using a three-staged cluster sampling method with strata, with the goal of understanding the composition and distribution of income, expenditures, and consumption in both urban and rural households [28,29].

To ensure the quality of the data the SCI follows several steps [30]; Interviews are used to collect expenditure data with the respondents completing the interview by a mix of recall and using documentation, non-contacted households are substituted, but not refusal households and non-response is reduced using more than one repeat visits. Missing values are imputed for some occasions; supplementary sources are not used to adjust estimates for under- or over-reporting; ethnic groups are excluded from data analysis.

These data have been used widely in other studies of health and household expenditures in Iran [19,27,31,32].

This study as a non-experimental research tried to compare income inequality and health inequality in Iran between 1984-2010. We used the pooled data from 1984–2010 includes 342,532 rural and 308,735 urban households; demographic characteristics of sample households are presented in Table 1.

**Table 1:** **Comparing variables in urban and rural households** - **Iran**: **1984**-**2010**

	**Urban**		**Rural**		**All**	
**Variable**	**No.**	**Percent**	**No.**	**Percent**	**No.**	**Percent**
**Observation**	308,735	47.41%	342,532	52.59%	651,267	100.00%
**Gender(Household’s Head)**						
Male	280,571	90.88%	303,895	88.72%	584,466	89.74%
Female	28,165	9.12%	38,636	11.28%	66,801	10.26%
**Education(Head of Household)**						
No-education	70,684	22.89%	164,149	47.92%	234,833	36.06%
Basic education	124,121	40.2%	121,522	35.48%	245,643	37.72%
Diploma	79,409	25.72%	35,005	10.22%	114,414	17.57%
Some college	34,521	11.18%	21,856	6.38%	56,377	8.66%
**Marital status(Household Head)**						
Married	265,565	86.02%	274,409	80.11%	539,974	82.91%
Widow	23,544	7.63%	40,513	11.83%	64,057	9.84%
Divorce	2,080	0.67%	2,260	0.66%	4,340	0.67%
Single	5,367	1.74%	12,217	3.57%	17,584	2.70%
Other	12,179	3.94%	13,133	3.83%	25,312	3.89%
**Other household’s characteristics**						
	No.	Mean (Std. Err.)	No.	Mean (Std. Err.)	No.	Mean (Std. Err.)
Household size	308,735	4.55 (0.004)	342,532	5.15 (0.004)	651,267	4.86 (0.003)
Age (Head of household)	308,735	46.03 (0.026)	342,532	48.45 (0.027)	651,267	47.03 (0.019)
Real Per-Capita Annual Income (Total Household Exp.; Rials)	308,735	40,384 (84)	342,532	18,823 (36)	651,267	29,044 (46)
Real Per-Capita Annual Total Health Exp. (Rials)	308,735	2,283 (26)	342,532	1,217 (11)	651,267	1,719 (14)

*Source*: *Authors*’ *calculations from Iran*’*s Household Expenditure and Income Survey*: *1984*–*2010*.

Notes:

1. Household per capita annual income (Per capita annual Total Household Exp.) and per capita Total Household Exp. for health was adjusted by Consumer Price Index (CPI) and considering 1997 = 100.

2. The gender distribution, literacy, marital status and income are significantly different between urban and rural areas. (P-values: 0.001).

3. Different Obs. for Total Health Exp. related to some missing values.

### Analysis

#### Adjustment for the composition of households

To account for the size and composition of each household, we converted household expenditures to per capita expenditures. We used a normalized household equivalence scale, including all households with heads at least 17 years of age and giving equal weight to all individuals in the household [33]. Total household expenditure is used as a proxy for household income, an approach recommended by Deaton as more reliable than self-reported income in surveys [34].

### Adjustment for health care and total expenditures

Household expenditures are adjusted over time to account for inflation, which varies in urban and rural areas. Per capita health care expenditure distribution in urban and rural areas was adjusted using the Consumer Price Index (CPI), as published by the Central Bank of Iran (CBI) [35] for urban areas and Statistical Center of Iran (SCI) for rural areas [36].

### Inequality analysis

#### Gini coefficient

The Gini Coefficient (GC) is the most common income inequality measure [33,37,38], with many desirable properties such as mean and population size independence, symmetry and Pigou-Dalton transfer sensitivity [33]. The coefficient ranges from zero (representing perfect income equality) to one (representing perfect income inequality). It is a function of the Lorenz curve, which depicts the distribution of income in a sample. Perfect equality is given by a straight line from the origin to (100,100), indicating that x% of the population earns x% of the cumulative income. In the case of complete inequality, in which the richest person earns all the income, the Lorenz curve would run along the x-axis with a right angle at (100.0) to terminate at (100,100). The greater the degree of inequality, the further the distance of the curve from the diagonal line running from the origin to the terminate point.

We focused on GC as a measure of inequality; it gave us the opportunity to compare inequalities in health expenditure with income inequalities (using total expenditure as proxy) over time and space. The cumulative frequency curve compares the distribution of total health care expenditures y_i_ with the cumulative percentage of population x_i_. The GC was calculated using the formula by Haughton (2009). Given a point (x_i_, y_i_), the GC will be:

(1) $$\mathrm{GC}=1-\sum_{i=1}^{N}\left(x_{i}-x_{i-1}\right)\left(y_{i}+y_{i-1}\right)$$

where *N* is the total number of observations. If *N* is equal to the interval on the x-axis, equation (2) can be simplified to:

(2) $$\mathrm{GC}\;=\;1\;‒\;\sum_{i=1}^{N}\left(y_{i}\;+\;y_{i‒1}\right)$$

### Progressivity analysis

Widely used in public finance, the Kakwani progressivity index (KPI) has been used to measure the degree of progressivity in health care finance [39]. The Kakwani index is given by *KPI* = *CI*-*GC* where *CI* is the concentration index for out-of-pocket health expenditures:

(3) $$\begin{array}{c} \mathrm{CI}\;=\;1+1/n+1/n^{2}\mu \left(HE_{1}+\mathrm{HE}2+L+HE_{n}\right)\\ \;n:\;\mathrm{SampleSize};\;\mathrm{HE}:\;\mathrm{HealthExpenditure},\;\mu \;=\;\mathrm{Mean} \end{array}$$

and GC is the Gini Coefficient for total household expenditure (income) [40]. The Kakwani index is equal to 1 in the most progressive system and −2 in the most regressive system [33].

## Results and discussion

### Results

In this study we used 27 years of data from the HIES, with 651,267 participating households in the total pooled sample. Table 1 compares mean absolute total expenditures and health care expenditures for urban and rural areas. Households in urban areas have higher levels of health expenditures than those in rural areas, but rural areas spend a larger proportion of their income on health.

According to the Iranian household structure around 90% of households in the sample are headed by males, around 23% of household heads have no formal schooling and approximately 90% of male household heads were married, compared to just 20% of female household heads.

Tables 2 and 3 report the inequality in the distribution of health care expenditures in urban and rural areas using the GC and KPI by year and NDP, respectively. It can be seen that inequality has persisted throughout the time period of analysis. From 1984 to 2010, inequality in health care expenditures (HE) was larger than the inequality for total expenditures; for example, mean GC in urban areas was 0.4448 for total expenditures and 0.7643 for total health expenditures.

**Table 2:** **Inequality measure for Per Capita real health expenditure by location**; **Iran**: **1984**-**2010**

**Year**	**Total health expenditure Gini coefficient**		**Total health expenditure Kakwani index**		**Total expenditure Gini coefficient**	
	**Urban**	**Rural**	**Urban**	**Rural**	**Urban**	**Rural**
1984	0.7510	0.7474	0.4553	0.4429	0.4875	0.4100
1985	0.7409	0.7199	0.4438	0.4118	0.4789	0.4008
1986	0.6940	0.6990	0.3904	0.3933	0.4667	0.4150
1987	0.8031	0.6536	0.5314	0.3454	0.4635	0.3844
1988	0.8074	0.7579	0.5380	0.4653	0.4525	0.3827
1989	0.7518	0.7531	0.4602	0.4568	0.4372	0.3826
1990	0.7310	0.7596	0.4355	0.4661	0.4148	0.4340
1991	0.7597	0.7953	0.4703	0.5144	0.4467	0.4537
1992	0.7856	0.7690	0.5039	0.4820	0.4195	0.4359
1993	0.7326	0.7618	0.4348	0.4736	0.4076	0.4190
1994	0.7634	0.7265	0.4717	0.4271	0.4239	0.4147
1995	0.7528	0.7477	0.4589	0.4538	0.4171	0.4221
1996	0.7569	0.7282	0.4659	0.4293	0.4332	0.4060
1997	0.7429	0.7247	0.4465	0.4261	0.4308	0.4107
1998	0.7453	0.7287	0.4497	0.4302	0.4276	0.4321
1999	0.7088	0.7200	0.4043	0.4172	0.4271	0.4185
2000	0.7134	0.6965	0.4107	0.3915	0.4241	0.4156
2001	0.7153	0.6913	0.4120	0.3862	0.4309	0.4080
2002	0.7121	0.6844	0.4084	0.3778	0.4361	0.4013
2003	0.6684	0.6832	0.3606	0.3765	0.4257	0.3928
2004	0.7706	0.7547	0.4784	0.4577	0.4202	0.4096
2005	0.7664	0.7267	0.4712	0.4244	0.4273	0.4070
2006	0.7673	0.7493	0.4735	0.4510	0.4422	0.4173
2007	0.7851	0.7235	0.4969	0.4198	0.4403	0.4099
2008	0.7693	0.7274	0.4759	0.4248	0.4129	0.3977
2009	0.7451	0.7395	0.4457	0.4389	0.4010	0.3970
2010	0.7452	0.7210	0.4458	0.4169	0.4016	0.3931
Total						
	**Urban**	**Rural**				
**Observations**	**308**,**735**	**342**,**532**	P-Value			
Gini Coeff.						
Total health expenditure	0.7643	0.7369	0.0364			
Total expenditure	0.4448	0.4159	0.0001			
Kakwani index						
Total health expenditure	0.4699	0.4363	0.0283			
Total expenditure	0.1692	0.1496	0.0993			

*Source*: *Authors*’ *calculations from Iran*’*s Household Expenditure and Income Survey*: *1984*–*2010*.

Notes:

1. *THEXP*: Total Health Expenditure; TEXP, Total Household Expenditure.

2. In the last three lines of this table we presented Gini Coefficient and KPI over the time (1984–2010).

**Table 3:** **Inequality measure for Per Capita real health expenditure in Iran by location in different periods in the National Development Plans** (**NDPs**)

**Gini Coefficient**						
**Total health expenditure**						
Years	NDPs	**Urban**	**St. Err**	**Rural**	**St. Err**	**P-Values**
1984-1989	Before NDPs	0.7580	(0.017)	0.7218	(0.016)	0.1597
1990-1994	First	0.7545	(0.010)	0.7624	(0.011)	0.6093
1995-1999	Second	0.7413	(0.009)	0.7299	(0.005)	0.2725
2000-2004	Third	0.7160	(0.016)	0.7020	(0.013)	0.5268
2005-2009	Fourth	0.7631	(0.006)	0.7312	(0.004)	0.0021
**Total expenditure**						
1984-1989	Before NDPs	0.4521	(0.007)	0.3868	(0.007)	0.0001
1990-1994	First	0.4150	(0.006)	0.4130	(0.006)	0.8282
1995-1999	Second	0.4169	(0.002)	0.4029	(0.005)	0.0352
2000-2004	Third	0.4199	(0.002)	0.3941	(0.004)	0.0003
2005-2009	Fourth	0.4168	(0.008)	0.3948	(0.004)	0.0292
**Kakwani index**						
**Total health expenditure**						
1984-1989	Before NDPs	0.4193	(0.019)	0.4699	(0.023)	0.1164
1990-1994	First	0.4726	(0.014)	0.4632	(0.013)	0.6360
1995-1999	Second	0.4313	(0.006)	0.4451	(0.011)	0.2980
2000-2004	Third	0.3979	(0.015)	0.4140	(0.019)	0.5242
2005-2009	Fourth	0.4293	(0.005)	0.4682	(0.008)	0.0024
**Total expenditure**						
1984-1989	Before NDPs	0.1835	(0.005)	0.1374	(0.004)	0.0000
1990-1994	First	0.1542	(0.005)	0.1602	(0.005)	0.4031
1995-1999	Second	0.1572	(0.002)	0.1508	(0.003)	0.1058
2000-2004	Third	0.1573	(0.002)	0.1427	(0.003)	0.0021
2005-2009	Fourth	0.1529	(0.005)	0.1415	(0.002)	0.0686

*Source*: *Authors*’ *calculations from Iran*’*s Household Expenditure and Income. Survey*: *1984*–*2010*.

While the analysis covers the time period 1984–2010, the first NDP was not adopted until 1989, and before that (1980–1988) Iran was engaged in the Iraq-Iran war. The GC on health expenditures peaks in urban areas towards the end of the war, and the urban KPI shows a lot of variation during this time as well. If we focus on the post-war period, health expenditure inequality remained high in both urban and rural areas until the mid-1990s, peaking in 1991 for rural and in 1992 for urban areas. Starting in the mid-1990s, inequality began to decline, a trend that continued until 2003 in both urban and rural areas.

Examining the trend in health expenditure inequalities across NDPs in Table 3, the lowest level of inequality is associated with the implementation of the third NDP. This does not necessarily indicate that the third NDP is solely responsible for improved inequality during this time, as the effect of the NDPs on inequality is likely somewhat lagged. This progress was not sustained in the fourth NDP, a time period that was associated with the most health inequality based on the GC in urban areas. The dynamics in rural areas were different, with the most post-war health inequality observed during the initial phase of the NDPs. This divergence in inequality trends in urban and rural areas may reflect the emphasis of the NDPs on addressing disparities in rural areas.

The KPI for health expenditure and total expenditure consistently indicated relative progressivity, with a greater degree of progressivity exhibited in health expenditures.

### Inequality in urban and rural areas

Table 2 compares the inequality in health care expenditures between urban and rural households. There is less inequality in rural areas, both in terms of health care expenditures and total expenditures.

Dramatic fluctuations can be seen when studying the trend of changes in health care expenditure inequality in rural areas of Iran.

**Figure 2:**
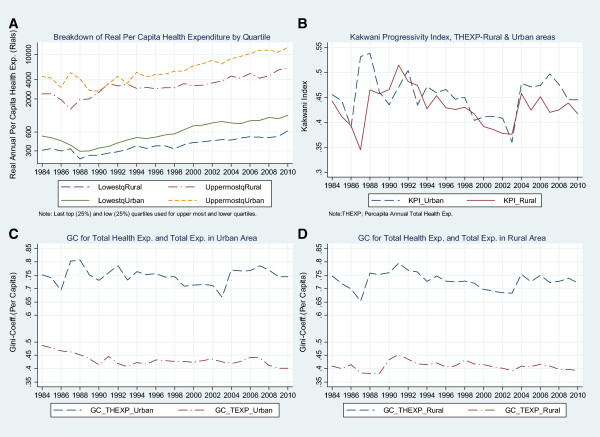
**Comparing changes in inequalities in total health exp.;Iran--1984-2010.**

Table 2 and Figure 2(B) document the progressivity of health care expenditures, using the KPI between 1984 and 2010. The KPI is positive in all years for both urban and rural areas, with means of 0.436 and 0.467 in rural and urban areas, respectively. The KPI index declined in both rural and urban areas from 1993 until 2003, and then rose sharply in 2004, much like the GC.

As presented in Figure 2(C and D), in 1988, 1992, 2004, 2007, there is a sharp increase in the Gini index for total health expenditure in urban areas and in 1988, 1991, 2004, 2006 and 2009, in rural areas, with the highest level observed in 1991 (0.795 for rural) and 0.785 in 2007 for urban areas; in the mid-2000s, the level of inequality reverts to, and eventually exceeds, war-time levels. Despite the introduction of universal coverage in 1995 [20] and changes in infrastructure that included expansion of the national health insurance coverage and implementation of poverty alleviation plans, the health expenditures GC during the time of the fourth NDP hinted at the instability of public policies in the health care system.

**Figure 3:**
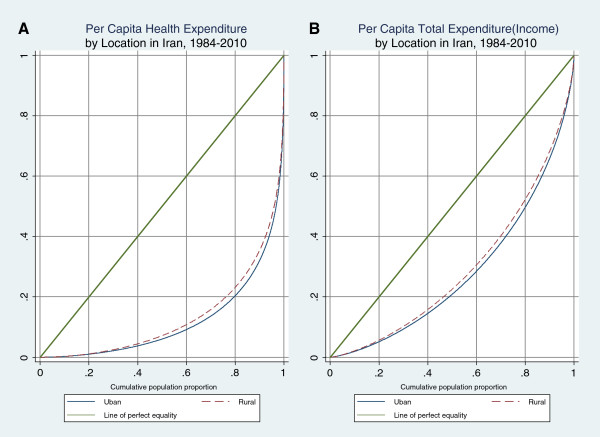
**Generalized Lorenz Curve for Per Capita Health Exp. and Total Exp. by Location in Iran,1984**–**2010.**

**Figure 4:**
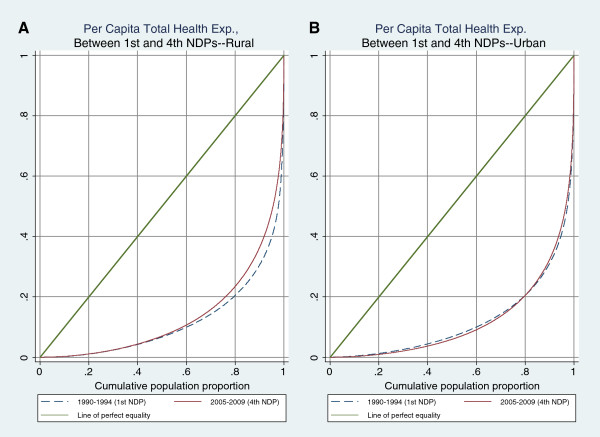
Comparing Generalized Lorenz Curve for Per Capita Health Exp. Between 1st and 4th NDPs in Iran.

Figure 3 shows the Lorenz curves for per capita adjusted health care expenditures and per capita income (total expenditures) in urban and rural areas. These curves reflect the inequalities presented in Tables 2 and 3. In Figure 3(A) and Figure 3(B) the rural Lorenz curve lies inside the urban Lorenz curve, indicating less inequality in health expenditures and income in rural areas.Figure 4 compares Lorenz curves for health care expenditures under the first and fourth NDPs for urban and rural areas. The rural curves suggest that during the NDPs inequality was reduced, while little or no change in inequality is observed during this time period in urban areas.

Using Table 3 to understand how the KPI varies across NDPs, the most progressivity was observed in rural areas before the NDPs and during the fourth NDP, while progressivity is highest in urban areas during the first NDP. There are no statistically significant differences between the KPIs in urban and rural areas in the first, second, and third NDPs, but the KPIs are significantly higher in rural areas during the fourth NDP.

### Inequality in male-headed and female-headed households

**Figure 5:**
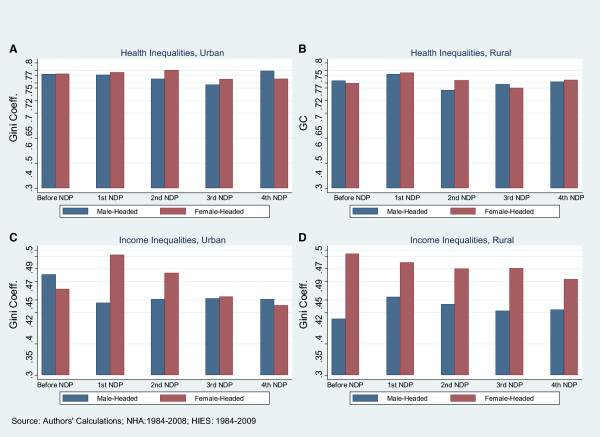
**Comparing Income Inequalities and Health Inequalities in Male**-**Headed and Female**-**Headed Households in Four NDPs in Urban and Rural Areas,Iran-1984-2009.**

As shown in Table 4 and Figure 5, for most of the last three decades inequality is slightly higher in households with female heads, and significantly higher in female-headed households in urban areas in terms of income. However, there is substantial variability in this relationship during this time period.Figure 5 documented income inequality and health inequality in male-headed and female-headed households in rural and urban areas before NDP and during four NDPs. Health expenditure inequality was lowest in urban areas during the years corresponding to the third NDP, after which inequality in male-headed households increased while it remained relatively stable among female-headed households.

**Table 4:** Inequality measure for per capita real health expenditures and total expenditure in male-headed and female-headed in urban and rural areas; Iran: 1984-2010

**Location**/**Inequality Measures**	**Urban**			**Rural**		
	**Male**-**headed**	**Female**-**headed**		**Male**-**headed**	**Female**-**headed**	
**Observations**	280,570	28,165		303,895	38,636	
	90.88%	9.12%		88.72%	11.28%	
**Gini coefficient**			p-value			p-value
Total health expenditure	0.7631	0.7681	0.3334	0.7352	0.7474	0.9696
Total Expenditure	0.44037	0.4826	0.0160	0.4122	0.4420	0.0573
**Kakwani index**						
Total health expenditure	0.4687	0.4732	0.7678	0.4346	0.4472	0.0551
Total expenditure	0.1661	0.1965	0.0183	0.1471	0.1678	0.9035

*Source*: *Authors*’ *calculations from Iran*’*s Household Expenditure and Income Survey*: *1984*–*2010.*

Notes:

1. Per Capita Total Health Expenditure and Total Expenditure were adjusted by considering CPI (1997 = 100).

2. GC and KPI reported inequalities in pool data between 1984–2010.

In both urban and rural areas, income inequalities in both male-headed and female-headed households were consistently lower than health inequalities. Towards the end of the analysis period, during the time corresponding with the third and fourth NDPs, the urban inequality gap closed in male- and female-headed households in terms of income. The same movement did not occur in rural areas, where inequality was persistently greater among female-headed households.

## Discussion

This paper provides a descriptive account of inequality in health care expenditures in Iran from 1984 to 2010; our findings are similar to other findings in different periods and policy areas. For example, Isfahani used HIES data from 1984 to 2005 and calculated the GC for total expenditures and concluded that, in spite of the implementation of different governmental policies, inequality and poverty remained significant problems in Iran [19]. Mahmodi used HIES data from 1989 to 1994 and used consumption as a proxy for income and concluded that not only was inequality in Iran very high^a^, but the gap between urban and rural areas was especially wide [31].

Our focus on health expenditures identified even greater inequality in this category than is evident in the economy as a whole. Iran has introduced several reforms to address inequalities in the health care system during this time period [16]. Perhaps the most important intervention was the introduction of health insurance as part of the second NDP in 1995 (which sought to decrease the financial burden of health care). The universal health insurance coverage law was ratified to extend coverage to everyone, giving priority to the poor, needy, and rural groups^b^. It is possible that the implementation of this law contributed to the decline in inequality observed during the third NDP.

In 2004, at the start of the fourth NDP, the Ministry of Welfare and Social Security (MWSS) was established to develop and improve the health insurance schemes. However, the trend of GC for health care expenditures suggests that the fourth NDP and MWSS were not able to achieve the objective of decreasing inequalities in the health system in Iran. While there could be a lag between the implementation of the policies and the impact they have on inequalities, the GC for health care expenditures remains higher than third –NDP levels through 2010.

While there were some improvements in inequality due to the NDPs, these appear to have been mostly temporary, and inequality in health expenditures surged during the latter half of the 2000s. This suggests that the strategies adopted as part of the NDPs were not able to sustainably reduce inequality in health care expenditures.

Our findings show that in spite of several major initiatives, such as the development of PHC networks and medical facilities in rural areas (in the first and second NDPs) and introduction of universal health insurance coverage in 1995 (in the second NDP), inequality has not declined. This is consistent with results from a study by Ibrahimipour et al., that noted that the lack of government participation was one of the main reasons for the failure to reach universal coverage of health insurance [16].

The distribution of catastrophic payments reflects the fairness in financing of health systems [41]. To understand the impact of NDPs on households, we considered the World Health Organization’s (WHO) measure for catastrophic payments (total health expenditure over total expenditure greater than or equal to 40%) [42]. According to this threshold, during the selected years on average 6.97% of the households in this study faced catastrophic levels of health care expenditures. The highest percentage of households met this threshold during the fourth NDP, including 15.12% of rural households and 16.63% of urban households. These rates peaked for urban and rural areas in the last two study years (2009 and 2010).

Our findings are similar to those presented in the 2007 World Bank Annual Report, which described funding for health in Iran as highly progressive with large out-of-pocket payments [43]. A study by Kavosi, which analyzed catastrophic payments in Iran from 2003 to 2008 also concluded that policy interventions did not decrease people’s contribution to health care expenditures during this period [10]. Furthermore, a study by Daneshkohan in Kermanshah reported that 22% of households in this region faced catastrophic levels of health expenditures in 2008 [44].

### Strengths and limitations

Access to reliable information in upper middle income countries such as Iran is one of the most significant obstacles to conducting this type of research. Despite the lack of official statistics in Iran, the data on household expenditures provide a reliable source of information for decision-making. The stability and reliability of self-reported household expenditure surveys are the main strength of these data [45]. However there are some well known issues with using household expenditure data such as inaccurate self-reporting and skewing of the data when wealthier households hire workers and supply them with food [46,47]. In spite of these drawbacks, consumption is considered to be a reliable indicator of household income in both developing and developed countries [45].

It is worth noting that this study does not attempt to identify the reasons why the NDPs succeeded or failed to reduce inequalities in health care expenditures. It only investigates the effects of the implemented policies on the inequality in health care expenditures of households. This is the first comprehensive study which assesses all of Iran’s developmental plans after the revolution and compares the objectives of the plans with the distribution of health expenditures among Iranian households.

## Conclusions

Over the past three decades Iran implemented four NDPs which introduced several reforms in the health care system. To understand the impact of NDPs on health expenditures of households we considered indicators of inequality (Gini coefficient) and out-of-pocket progressivity (Kakwani index).

Comparing the trend of out-of-pocket payments in Iran showed that it consistently represented more than half of total health care expenditures, with no significant change during the course of the NDPs. A comparison of inequality in health care expenditures over time showed that inequality was persistent, and was higher in urban areas.

Considering these effects, policymakers need to consider revising existing policies to improve equality in Iran. It seems that a revision of social protection, social safety nets, and health insurance schemes would be options for decreasing inequalities in health care expenditures in this area. Other mechanisms, such as ensuring better coverage for individuals with certain chronic diseases, the elderly, and other vulnerable groups may also improve the current situation.

The results from this paper may be relevant to other countries with similar health care systems, and those countries where oil is the main source of government revenue such as Algeria and Venezuela, as well as other countries in the region such as Turkey and Saudi Arabia which have similar socio-demographic profiles.

### Endnotes

^a^Despite an increase in Iran’s revenue from 1990 to 1992—due to the sudden oil price hike—the inequality gap persisted in rural areas in late 1994. The increase in 1998 can be attributed to the economic shock in the country due to the impact of the economic reform in Iran.

^b^Before the MSIO was established civil servants were the only ones covered by public health insurance.

## Abbreviations

CBI: Central Bank of Iran; CPI: Consumer Price Index; GC: Gini Coefficient; GDP: Gross Domestic Product; HIES: Household Income and Expenditure Survey; KPI: Kakwani progressivity index; MSIO: Medical Services Insurance Organization; MOHME: Ministry of Health and Medical Education; NDPs: National Development Plans; NGOs: Non-governmental organizations; PHC: Primary health care; SSO: Social Security Organization; SCI: Statistical Center of Iran; MWSS: The Ministry of Welfare and Social Security.

## Competing interests

The authors’ declare that they have no competing interests.

## Authors’ contributions

HZ participated in the design and concept of the study, carried out the statistical analysis, and drafted the manuscript. AT participated in the design, concept of the study and drafting of the manuscript. JD, GG and MG participated in the interpretation of the results and revision of the manuscript. All authors read and approved the final manuscript.
